# Unmasking Hemophagocytic Lymphohistiocytosis After Minimal Tumor Necrosis Factor-Alpha (TNF-α) Blockade: An Interplay of Contributing Elements

**DOI:** 10.7759/cureus.101174

**Published:** 2026-01-09

**Authors:** Shaivya Pathak

**Affiliations:** 1 Internal Medicine, East Carolina University, Greenville, USA

**Keywords:** adalimumab, drug-induced hlh, hemophagocytic lymphohistiocytosis, macrophage activation syndrome, tnf-alpha inhibitor

## Abstract

Presented is a case of hemophagocytic lymphohistiocytosis (HLH) in a 25-year-old African American female patient with Behçet disease and seronegative rheumatoid arthritis who developed fever, nausea, vomiting, and diarrhea 14 days after her first adalimumab dose. The diagnosis was established based on clinical and laboratory parameters meeting five of eight HLH-2004 criteria. The temporal relationship between drug initiation and symptom onset, along with prior improvement in arthritis symptoms, favored drug-induced HLH over macrophage activation syndrome. The patient was successfully treated with high-dose dexamethasone monotherapy per the HLH-2004 protocol, with etoposide deferred given the suspected drug-induced etiology and normal NK activity, achieving clinical improvement within 48 to 72 hours. This case demonstrates that HLH can occur after minimal tumor necrosis factor-alpha inhibitor exposure, likely through idiosyncratic mechanisms potentiated by multiple autoimmune conditions.

## Introduction

Tumor necrosis factor-alpha (TNF-α) inhibitors have revolutionized the treatment of inflammatory arthritides and autoimmune conditions, demonstrating remarkable efficacy in controlling disease activity and preventing structural damage [1]. However, paradoxically, these immunomodulating agents have been increasingly recognized as potential triggers for hemophagocytic lymphohistiocytosis (HLH), a rare but life-threatening syndrome of excessive immune activation and tissue destruction.

HLH is characterized by aberrant activation and proliferation of macrophages and cytotoxic T lymphocytes, leading to a hyperinflammatory state with uncontrolled cytokine production. The clinical presentation typically includes persistent fever, cytopenias, hepatosplenomegaly, hyperferritinemia, and multi-organ dysfunction [2]. While primary HLH occurs due to genetic mutations affecting cytotoxic function, secondary HLH can be triggered by infections, malignancies, or increasingly recognized, by immunosuppressive therapies including biologics [3].

The relationship between TNF-α blockade and HLH development appears paradoxical, as TNF-α plays a central role in the inflammatory cascade underlying HLH pathogenesis. Several mechanisms have been proposed to explain this phenomenon [1]. 

Adalimumab, a fully human monoclonal antibody against TNF-α, is widely used for treating rheumatological conditions, with a generally favorable safety profile. However, post-marketing surveillance and case reports have documented rare instances of HLH occurring within days to months after adalimumab initiation [4]. The diagnosis is particularly challenging in patients with underlying rheumatic diseases, as HLH shares clinical and laboratory features with macrophage activation syndrome (MAS), a related hyperinflammatory condition that represents the rheumatologic spectrum of secondary HLH. Distinguishing between infection-triggered HLH, MAS, and drug-induced HLH in patients receiving biologics requires careful clinical evaluation and often empirical management given the condition's high mortality rate.

Presented is a case of a 25-year-old African American female patient with a complex rheumatologic history who developed clinical and laboratory features consistent with HLH 14 days after her first dose of adalimumab. This case highlights the importance of maintaining high clinical suspicion for HLH in patients presenting with systemic inflammatory symptoms following TNF-α inhibitor initiation.

## Case presentation

A 25-year-old African American woman with a past medical history of childhood-onset Behçet disease and seronegative rheumatoid arthritis presented to the emergency department with a four-day history of gastrointestinal symptoms. The patient had been started on adalimumab (Humira) 40 mg subcutaneously 14 days prior to presentation for management of her rheumatoid arthritis, following completion of a corticosteroid taper a month prior.

Four days prior to admission, the patient developed low-grade fevers associated with progressive nausea and intractable vomiting, limiting her ability to maintain oral intake. She simultaneously developed watery, non-bloody diarrhea without associated abdominal cramping. Additional symptoms included frontal headache and generalized malaise. She denied night sweats, unintentional weight loss, recent travel, sick contacts, or consumption of questionable foods. The patient reported no prior similar episodes and specifically denied any current joint pain, morning stiffness, oral or genital ulcers, or other manifestations of her underlying rheumatologic conditions.

**Figure 1:**
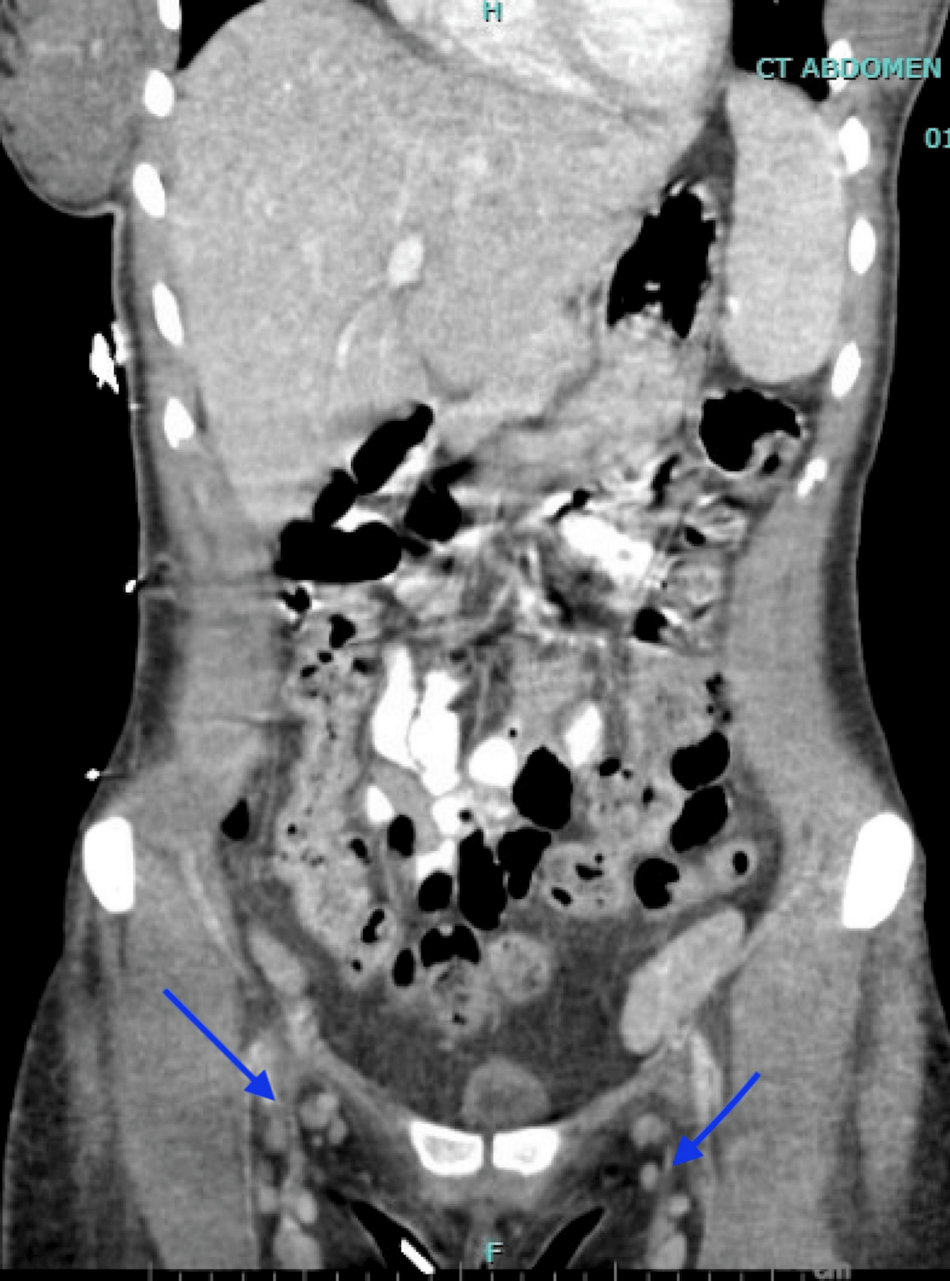
CT Abdomen and Pelvis with Intravenous Contrast in the Coronal Plane Blue arrows show numerous bilateral subcentimeter inguinal lymph nodes. CT: Computed Tomography

**Figure 2:**
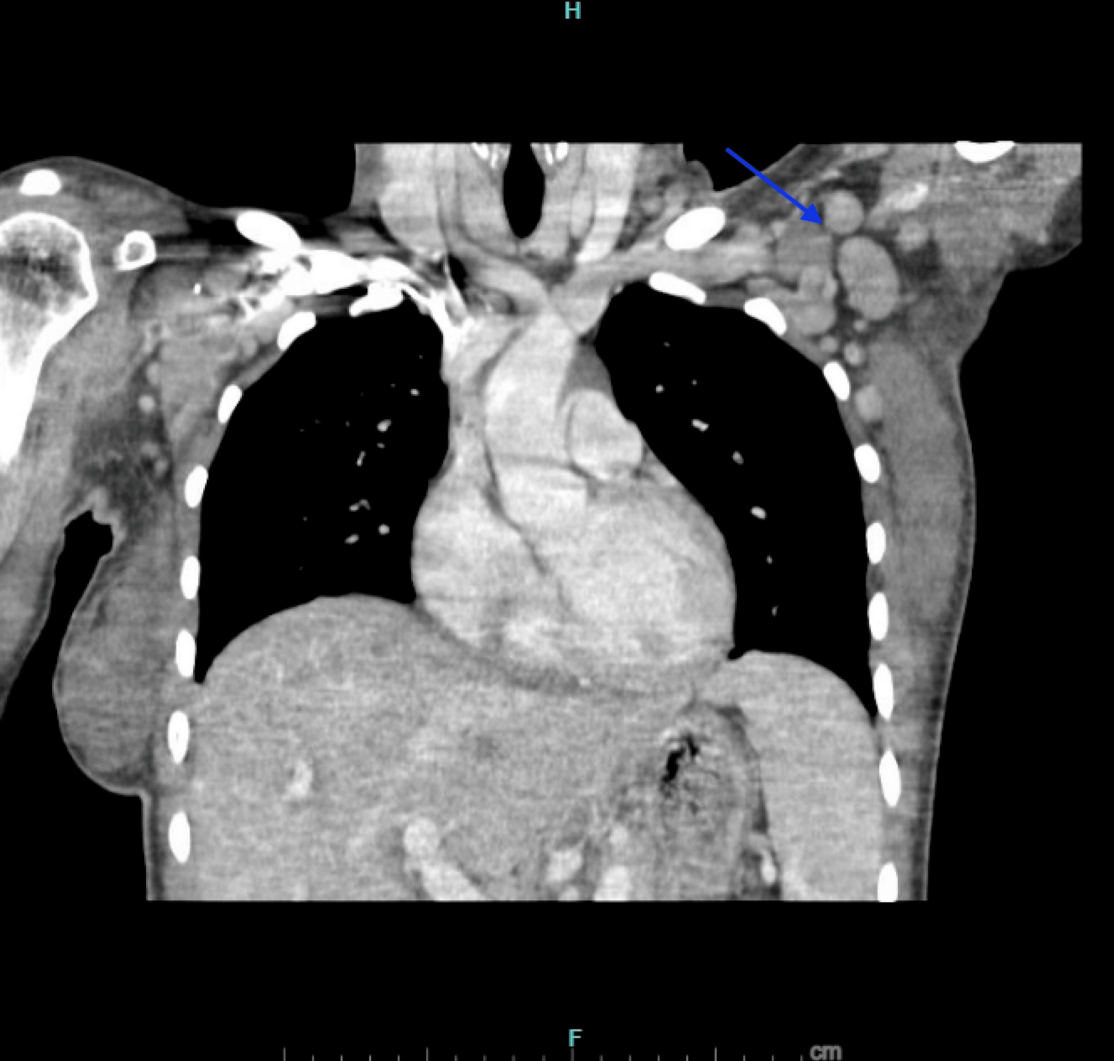
CT Chest with Intravenous Contrast in the Coronal Plane The blue arrow shows lymphadenopathy in the left axilla. CT: Computed Tomography

**Figure 3:**
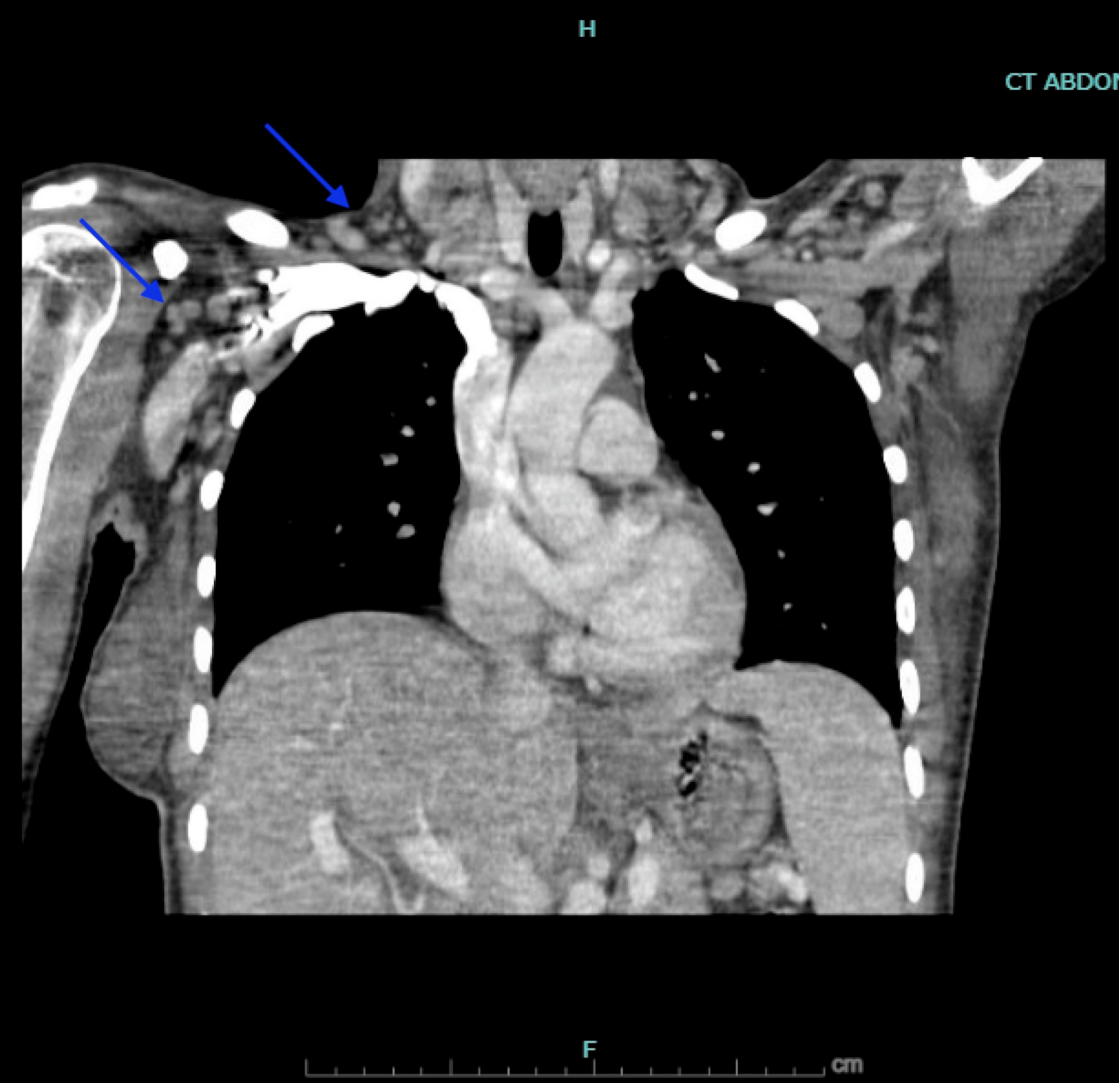
CT Chest with Intravenous Contrast in the Coronal Plane Blue arrows show numerous subcentimeter right axillary and supraclavicular lymph nodes. CT: Computed Tomography

On presentation to the emergency department, vital signs were notable for fever of 39.5°C (103.1°F), blood pressure 106/54 mmHg, tachycardia with pulse 115 beats per minute, and a respiratory rate of 20 breaths per minute with oxygen saturation 97% on room air. Physical examination revealed an ill-appearing young woman in mild distress. She was alert and oriented but appeared fatigued. Lab values are presented in Table 1. Imaging was pursued, a CT abdomen, pelvis, and chest with intravenous contrast showed supraclavicular, axillary, and inguinal nodes (Figures 1-3). Lymphadenopathy was characterized as numerous nodes of normal to minimally enlarged caliber, a nonspecific finding consistent with reactive lymphadenopathy. Abdominal ultrasonography demonstrated normal splenic dimensions.

**Table 1:** Laboratory Values

Test Category	Test Name	Result	Normal Range	Interpretation/Notes
Complete Blood Count with Manual Differential	WBC (White Blood Cell Count)	12.97	4.5 - 11.0 ×10^9/L	Leukocytosis
	RBC (Red Blood Cell Count)	3.7	4.2 - 5.4 ×10^12/L	Anemia
	Hemoglobin	9.1	12.0 - 15.5 g/dL	Anemia
	Hematocrit	29.6	36 - 48%	Anemia
	MCH (Mean Corpuscular Hemoglobin)	24.6	27 - 33 pg	Hypochromic
	MCHC (Mean Corpuscular Hemoglobin Concentration)	30.7	33 - 36 g/dL	Hypochromic
	RDW (Red Cell Distribution Width)	15.6	11.5 - 14.5 %	Suggests Variability in RBC Size
	Platelet Count	140	150 - 450 ×109/L	Thrombocytopenia
	MPV (Mean Platelet Volume)	10.7	7.5 - 10.5 fL	Suggests Production of Larger, Younger Platelets
	Vacuolization	Present	Absent	Abnormal
	Neutrophils (%)	82	40 - 75 %	Neutrophilia
	Bands (%)	10	0 - 5 %	Abnormal
	Lymphocytes (%)	4	20 - 45 %	Lymphopenia
	Myelocytes (%)	1	0 %	Abnormal
	Absolute Neutrophils/Bands (#)	11.93	1.8 - 8.0 ×109/L	Absolute Neutrophilia
	Absolute Lymphocytes (#)	0.52	1.0 - 4.8 ×109/L	Absolute Lymphopenia
	Absolute Myelocytes (#)	0.13	0 ×109/L	Presence of Immature Cells
Comprehensive Metabolic Panel	Sodium	125	135 - 145 mmol/L	Hyponatremia
	Chloride	94	98 - 107 mmol/L	Hypochloremia
	CO2	20	22 - 30 mmol/L	Metabolic Acidosis/Low Bicarb
	Calcium	8.3	8.5 - 10.5 mg/dL	Hypocalcemia
	Protein, Total	9.2	6.0 - 8.3 g/dL	Hyperproteinemia
	Albumin	3.1	3.5 - 5.0 g/dL	Hypoalbuminemia
	SGOT (AST)	445	8 - 48 U/L	Elevated Liver Enzyme
	SGPT (ALT)	87	7 - 55 U/L	Elevated Liver Enzyme
	Globulin (Calc'd)	6.1	2.0 - 3.9 g/dL (Calculated)	Calculated Globulin is High
	A:G Ratio	0.51	1.1 - 2.5	Low (Due to High Globulin and Low Albumin)
Coagulation/Renal/Cardiac	Fibrinogen	171	200 - 400 mg/dL	Hypofibrinogenemia
	Creatinine	0.96	0.5 - 1.1 mg/dL	Normal
	BUN	15	7 - 20 mg/dL	Normal
	Troponin I	< 0.04	< 0.04 ng/mL	Normal
	Lactate Dehydrogenase (LDH)	2023	140 - 280 U/L	Markedly Elevated
	Triglycerides	283	< 150 mg/dL	Hypertriglyceridemia
Infectious Disease	Respiratory Syncytial Virus	Negative	Negative	
	Influenza A by PCR	Negative	Negative	
	Influenza B by PCR	Negative	Negative	
	SARS-Coronavirus-2 PCR	Negative	Negative	
	HEP B CORE AB, IGM	Negative	Negative	
	Hepatitis A: HAV-IGM	Negative	Negative	
	Hepatitis B SURF AG	Negative	Negative	
	Hepatitis C Ab	Negative	Negative	
	Blood Culture (Bacteria)	Negative	Negative	
	Blood Culture (Fungal)	Negative	Negative	
	Urine Culture	Negative	Negative	
	Cytomegalovirus (CMV) DNA PCR	Negative	Negative	
	Epstein-Barr Virus (EBV) DNA PCR	Negative	Negative	
	Urine Gonorrhea/Chlamydia NAAT	Negative	Negative	
Inflammatory/Infectious Markers	C-Reactive Protein (CRP)	0.76	< 0.60 mg/dL	Elevated
	Erythrocyte Sedimentation Rate (ESR)	> 130	< 20 mm/hr	Markedly Elevated
	Ferritin	> 40,000	20 - 250 ng/mL (Female)	Extreme Hyperferritinemia
Immunologic/Cytokine	Soluble Interleukin-2 Receptor (sCD25)	4245	< 1,033 U/mL	Markedly Elevated
	NK Cell Activity	1.3	> 1.0 Lytic Units (LU)	Normal

Hematology consultation on day 2 of admission confirmed the diagnosis of probable HLH based on meeting at least five of eight HLH-2004 criteria, which included fever, cytopenias, hypofibrinogenemia, high ferritin, and high sCD25 (Table 1). Given the patient's underlying rheumatoid arthritis and recent adalimumab exposure, the differential included both secondary HLH and macrophage activation syndrome. The patient was started on a seven-week dexamethasone taper per the HLH-2004 protocol. During the initial 48 to 72 hours of steroid therapy, the patient's clinical status began to improve. Her fevers decreased in frequency and intensity, and her gastrointestinal symptoms resolved completely, allowing for adequate oral intake. Following multidisciplinary discussion, rheumatology recommended holding adalimumab given its probable role as the triggering agent for HLH. Plans were made for close outpatient rheumatology follow-up to reassess disease activity and determine alternative treatment strategies for her rheumatoid arthritis once the acute HLH resolved. The patient was discharged home after three days of admission.

## Discussion

This case represents a rare presentation of HLH occurring after a single dose of adalimumab in a patient with multiple autoimmune conditions. MAS is a term frequently used to describe secondary HLH occurring in the context of rheumatic diseases, most commonly systemic juvenile idiopathic arthritis and adult-onset Still's disease [5]. While MAS and HLH share overlapping clinical and laboratory features, the underlying trigger differs significantly. MAS typically arises during periods of active, uncontrolled rheumatic disease, whereas drug-induced HLH occurs as an idiosyncratic reaction to a pharmacologic agent. In this case, the temporal relationship with adalimumab initiation (14 days), the improvement in arthritis symptoms prior to symptom onset, and the absence of active joint inflammation, oral ulcers, or other rheumatic manifestations at presentation favored drug-induced HLH over MAS from active rheumatic disease. This distinction has critical implications for both acute management and long-term treatment planning, as drug-induced HLH typically responds to discontinuation of the offending agent and corticosteroids, while MAS requires treatment of the underlying rheumatic flare. Several interconnected mechanisms may explain this severe inflammatory response.

TNF-drug immune complex formation

**Figure 4:**
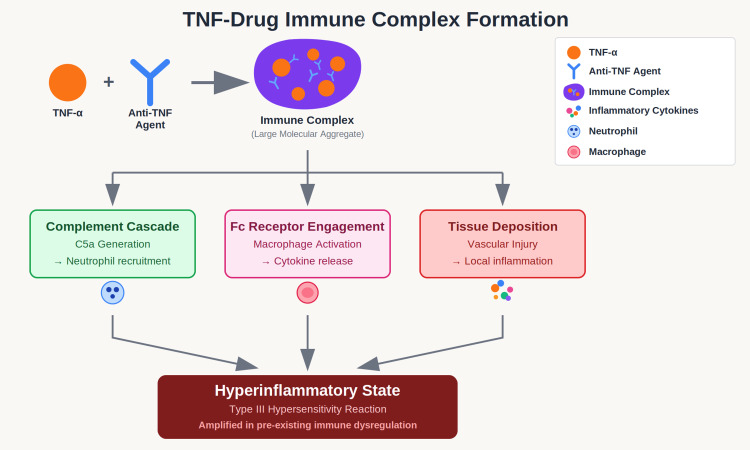
TNF-Drug Immune Complex Formation Proposed mechanism by which TNF-α and drug immune complex formation can further exacerbate a hyperinflammatory state. TNF-α: Tumor Necrosis Factor

Anti-TNF agents can form immune complexes with soluble TNF-α, creating large molecular aggregates that may paradoxically trigger inflammatory responses [6]. In patients with multiple autoimmune conditions, dysregulated antibody production and altered complement activation may amplify the inflammatory potential of these complexes. These TNF-drug complexes activate complement cascades, leading to C5a generation and subsequent neutrophil recruitment to sites of inflammation. Furthermore, these complexes engage Fc receptors on macrophages, triggering the release of inflammatory cytokines that perpetuate the hyperinflammatory state. The deposition of these immune complexes in tissues can cause local inflammation and vascular injury, while also stimulating type III hypersensitivity reactions in genetically susceptible individuals (Figure 4). This cascade of events may be particularly pronounced in patients with pre-existing immune dysregulation where the normal regulatory mechanisms that would limit such responses are already compromised [7,8]. 

Paradoxical autoimmune inflammation

Another critical mechanism involves interferon-alpha upregulation, as TNF-α normally suppresses plasmacytoid dendritic cell production of IFN-α. When TNF blockade releases this inhibition, the resulting increased IFN-α levels can trigger inflammatory cascades central to HLH pathogenesis [9].

**Figure 5:**
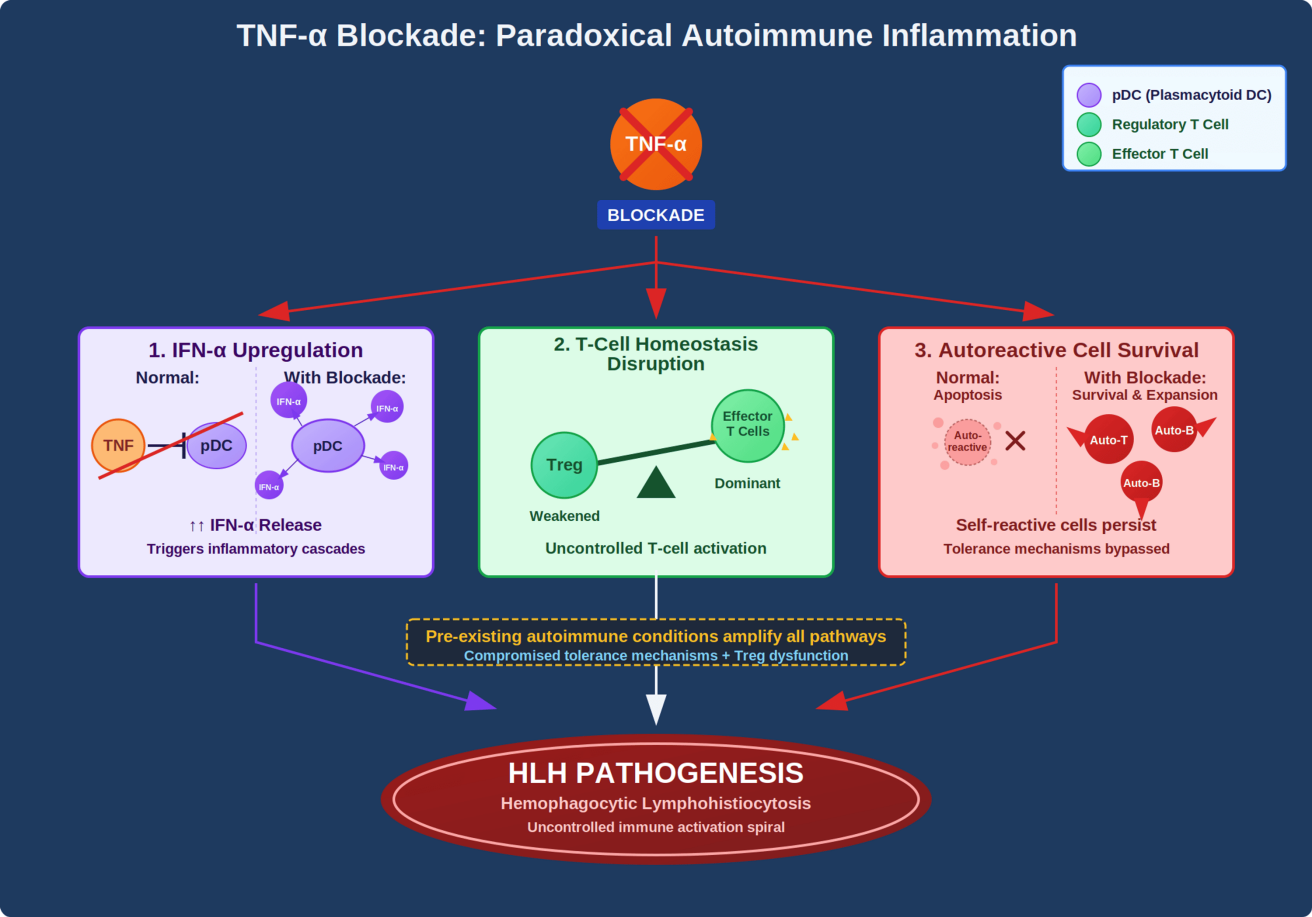
Paradoxical Autoimmune Inflammation Three different pathways through which TNF-α can paradoxically contribute to the pathogenesis of HLH. TNF-α: Tumor Necrosis Factor; HLH: Hemophagocytic Lymphohistiocytosis; IFN-α: Interferon-Alpha

Additionally, TNF-α inhibition disrupts T-cell homeostasis by altering the delicate balance between regulatory T cells (Tregs) and effector T cells. In patients with multiple autoimmune diseases, pre-existing Treg dysfunction may make them particularly vulnerable to this imbalance, precipitating the uncontrolled T-cell activation characteristic of HLH [10]. This disruption is compounded by the effect on autoreactive lymphocyte survival, as TNF-α normally promotes apoptosis of these self-reactive cells. Its blockade allows the survival and expansion of self-reactive T and B cells, a particularly problematic outcome in patients with existing autoimmune conditions where central and peripheral tolerance mechanisms are already compromised [11]. The convergence of these effects creates an environment where autoimmune inflammation can spiral out of control, even after a single exposure to TNF-α blockade (Figure 5).

Cytolytic pathway suppression

**Figure 6:**
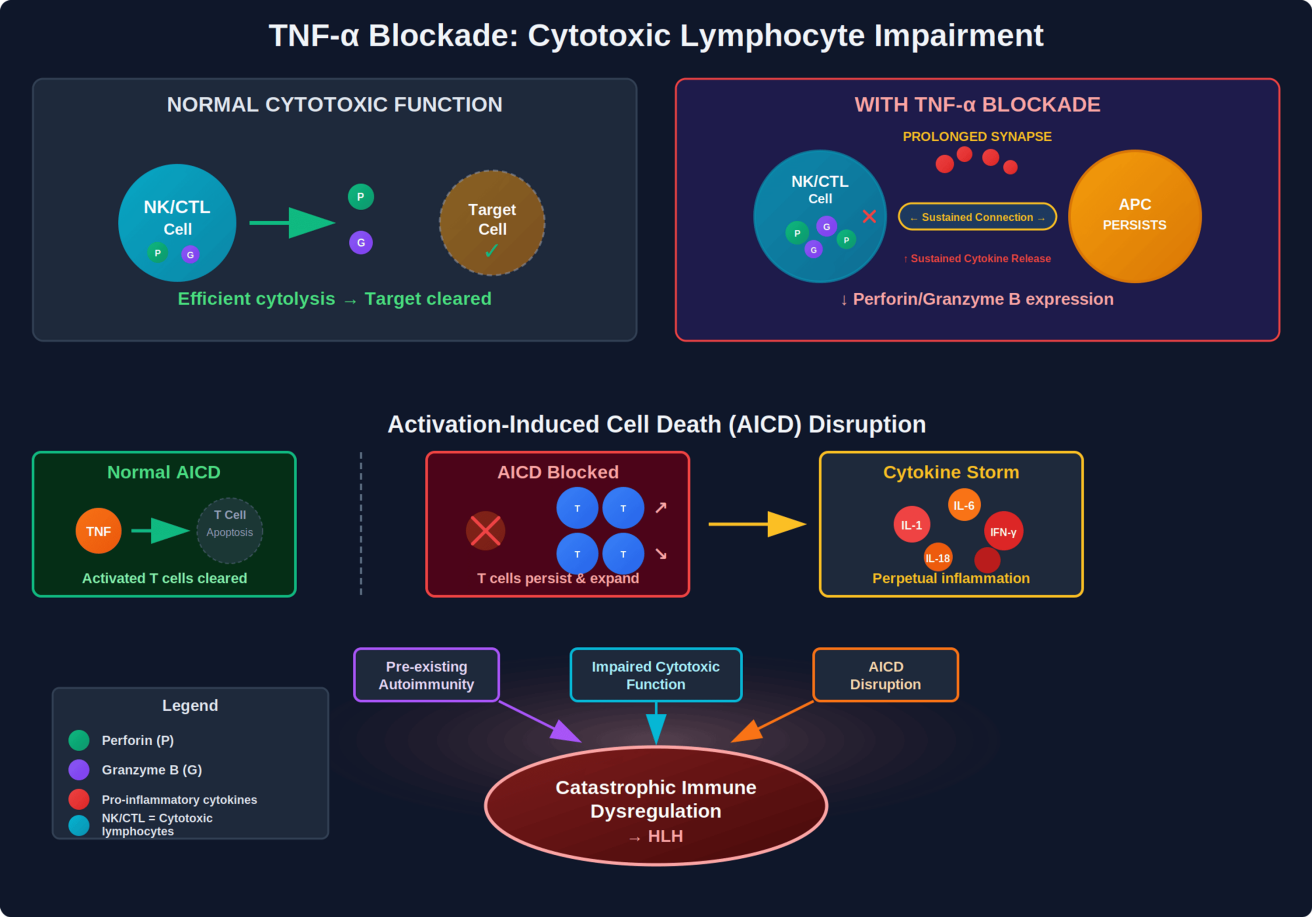
TNF-α Blockade-Induced Cytotoxic Lymphocyte Impairment Cellular and molecular pathways through which TNF-α blockade induced cytokine production can contribute to immune dysregulation and HLH pathogenesis. TNF-α: Tumor Necrosis Factor; HLH: Hemophagocytic Lymphohistiocytosis; AICD: Activation-Induced Cell Death

TNF-α plays a complex role in cytotoxic lymphocyte function, and its inhibition can impair critical cytolytic pathways through multiple mechanisms (Figure 6). Although our patient demonstrated normal NK cell activity at baseline, TNF-α blockade may acutely impair perforin and granzyme B expression, thereby reducing cytotoxic efficiency. This impairment leads to prolonged immunological synapses between cytotoxic cells and antigen-presenting cells, driving the sustained cytokine production characteristic of HLH [12,13].sThe disruption extends to activation-induced cell death (AICD) pathways, as TNF-α normally contributes to AICDs of lymphocytes following immune responses. When this pathway is blocked, activated T cells persist rather than undergoing appropriate apoptosis, perpetuating the inflammatory response seen in HLH [14].

Treatment considerations: role of etoposide

The decision to defer etoposide, a cornerstone of the HLH-2004 protocol, reflected careful consideration of the underlying etiology and patient-specific factors. Etoposide, a topoisomerase II inhibitor, is primarily indicated for primary (familial) HLH, EBV-associated HLH, malignancy-associated HLH, and refractory cases not responding to initial therapy [15].

In our case, several factors supported withholding etoposide from the treatment regimen. The finding of normal NK cell activity strongly argued against familial HLH, where NK function is characteristically impaired due to genetic defects in cytotoxic machinery [16]. Additionally, secondary HLH due to medication typically responds favorably to drug discontinuation and corticosteroids alone, without requiring cytotoxic therapy [17]. The absence of any evidence of underlying lymphoma or other malignancies further supported this conservative approach, as these conditions would typically necessitate etoposide therapy. This approach aligns with recent expert recommendations suggesting risk-stratified treatment for secondary HLH, where etoposide is reserved for severe or refractory cases rather than used uniformly [18]. The successful response to dexamethasone monotherapy in our patient validated this conservative approach.

## Conclusions

The complex pathophysiology involving TNF-drug immune complexes, paradoxical autoimmune inflammation, and cytolytic pathway suppression underscores the intricate balance required for immune homeostasis. In patients with pre-existing immune dysregulation, even a single perturbation of this balance can trigger a cascade of uncontrolled inflammation. This understanding emphasizes the need for careful patient selection and monitoring when initiating biologic therapies, particularly in those with multiple autoimmune conditions. While TNF-α inhibitors remain invaluable tools in managing inflammatory arthropathies, awareness of rare complications like drug-induced HLH is essential for early recognition and intervention.
